# Diagnostic significance of neutrophil to lymphocyte ratio in endometriosis: a systematic review and meta-analysis

**DOI:** 10.1186/s12905-023-02692-7

**Published:** 2023-11-07

**Authors:** Fatemeh Tabatabaei, Hossein Tahernia, Arshin Ghaedi, Aida Bazrgar, Shokoufeh Khanzadeh

**Affiliations:** 1 Department of Obstetrics and Gynaecology, School of Medicine, Tabriz University of Medical Sciences, Tabriz, Iran; 2 Department of Gynaecologic Laparoscopic Surgeries, Al-Zahra Hospital, Tabriz University of Medical Sciences, Tabriz, Iran; 3 Ranfsanjan University of Medical Science, Rafsanjan, Iran; 4 Student Research Committee, School of Medicine, Shiraz University of Medical Sciences, Shiraz, Iran; 5 Trauma Research Center, Shahid Rajaee (Emtiaz) Trauma Hospital, Shiraz University of Medical Sciences, Shiraz, Iran; 6 Student Research Committee, Tabriz University of Medical Sciences, Tabriz, Iran

**Keywords:** Neutrophil to lymphocyte ratio, NLR, Endometriosis, Meta-analysis

## Abstract

**Background:**

The purpose of this systematic review and meta-analysis was to compile existing evidence on the significance of the NLR in predicting endometriosis in order to aid clinical decision-making and outcomes.

**Methods:**

We searched ProQuest, Web of Science, and PubMed for related studies published before January 2, 2023. Standardized mean difference (SMD) with a 95% confidence interval (CI) was reported for each outcome. Because a significant level of heterogeneity was found, we used the random-effects model to calculate pooled effects. We used Newcastle-Ottawa Scale (NOS) for quality assessment.

**Results:**

Overall, 18 article with were included in the analysis. A random-effect model revealed that patients with endometriosis had elevated levels of NLR compared to healthy controls (SMD = 0.79, 95% CI = 0.33 to 1.25, *P* < 0.001). Patients with endometriosis had elevated levels of NLR compared to those with other benign tumors (SMD = 0.85, 95% CI = 0.17 to 1.53, *P* = 0.014). In addition, NLR level of patients with stage III and IV endometriosis was not different from that of patients with stage I and II endometrioma (SMD = 0.30, 95% CI = -0.14 to 0.74, *P* = 0.18). However, NLR level was not different between endometriosis patients with and without peritoneal lesions (SMD = -0.12, 95% CI = -0.34to 0.10, *P* = 0.28), between patients with and without endometrioma (SMD = 0.20, 95% CI = -0.15 to 0.55, *P* = 0.26) and between endometriosis patients with and without deep lesions (SMD = 0.04, 95% CI = -0.20 to 0.28, *P* = 0.72). The pooled sensitivity of NLR was 0.67 (95% CI = 0.60–0.73), and the pooled specificity was 0.68 (95% CI, 0.62–0.73).

**Conclusions:**

NLR might be utilized in clinics as a possible predictor to help clinicians diagnose endometriosis in affected women.

## Background

The existence of endometrial tissue outside the uterine cavity is known as endometriosis. In the general population, endometriosis is thought to affect 10 to 15% of people, and it may affect up to 70% of women who have chronic pain in the pelvic region [1–3]. Endometriosis patients often have infertility and dysmenorrhea, drastically reducing their quality of life [2]. Yet, there is not a uniform approach in dealing with these symptoms, and the illness can recur even after undergoing appropriate surgical procedures or using medications [4, 5]. Notably, surgery can be conceptualized as a cytoreductive approach aimed at eradicating the condition, yet endometriosis recurrence remains plausible. Conversely, medical treatment works by suppressing endometriosis. In a similar yet distinct manner, if medical treatment is stopped, the illness might become active again [6].

However, even when assisted reproductive technology (ART) enables a pregnancy, the profound emotional distress associated with infertility persists, particularly when considering the challenging hormonal therapies women undergo [7, 8]. Gynecological ailments like endometriosis might contribute to the emergence of anxiety and depression in these women [9]. Nonetheless, the comprehensive ART route can amplify substantial psychological distress for both genders, particularly in scenarios marked by iterative disappointments [10]. The particular medical conditions that hinder the occurrence of spontaneous pregnancy can serve as targeted psychological risk elements, shaping the paths of individual and couple development. These challenges encompass issues like anxiety and depressive disorders, and tendencies toward self-blame [11, 12]. Additionally, the sexual life of women is adversely affected [13].

There is significant patient diversity regarding illness phenotype and related symptom severity. Although abnormally high levels of estrogen and chronic inflammation are prominent symptoms of endometriosis, the underlying cause of the illness remains unclear [1]. This is due to the disease's multifactorial and complex character, which has already been linked to hormonal, genetic, immunological, and environmental variables [14]. Understanding the pathogenesis of endometriosis and attempting to avoid it, is one of the primary issues of contemporary gynecology. The most generally acknowledged explanation of endometrial lesion development to date is that while menstruation, cells and tissues of endometrium retract into the fallopian tubes and attach to the structures of the pelvic, resulting in discomfort, fibrosis, and also an inflammatory response [15–18]. Numerous inflammatory indicators, such as interleukins, neutrophils, and neutrophil to lymphocyte ratio (NLR), are raised in severe endometriosis because of the chronic inflammatory course of the disease [19–21]. In particular, the NLR has been suggested as a straightforward and practical prognostic and diagnostic biomarker for several illnesses [22–39]. Without early management and a precise diagnosis, endometriosis may become an emerging gynecological problem and cause infertility and dysmenorrhea.

As new studies on NLR's utility in endometriosis accumulate, a systematic review to assist clinical decision-making is necessary. The goal is to comprehend what an increased ratio means for a patient with endometriosis in order to implement early therapies and enhance outcomes. As far as we know, there are no systematic reviews on using NLR in this setting in the available literature. The purpose of this systematic review and meta-analysis was to compile existing evidence on the significance of the NLR in predicting endometriosis in order to aid clinical decision-making and outcomes.

## Materials and methods

### Search strategy, eligibility criteria, and study selection

The keywords "endometriosis," "NLR," and "neutrophil to lymphocyte ratio" were used in combination to perform a thorough search of articles in online databases like Web of Science, PubMed, and ProQuest from their establishment before January 2, 2023. Two researchers independently evaluated the relevancy of abstracts. Then, they separately reviewed the full texts of the relevant articles. The following were the requirements for eligibility: (1) evaluation of NLR; (2) comparison of endometriosis-affected and unaffected human participants; (3) cross-sectional or case–control design; and (4) full-text publications.

The references of the retrieved publications and reviews were also examined to discover further related articles. A third researcher arbitrated any disagreements that arose between the reviewers. To rate the quality of each research, we employed the Newcastle–Ottawa scale (NOS). The NOS assessed the following elements: selection of the cohort, and comparability of groups.

### Statistical analysis

Standardized mean differences (SMDs) were employed to compare NLR levels between healthy controls and patients with endometriosis and also to create forest plots of continuous data. Statistical significance was defined as *p* < 0.05, and 95% confidence intervals (CIs) were reported. The mean and standard deviation values were extrapolated from the median and IQR/range values in three studies, as previously explained [40].

The Q statistic was used to assess the heterogeneity of SMD across studies (significance level at *p* < 0.10). Additionally, the I^2^ statistic, a quantitative indicator of study inconsistency, was calculated. I^2^ values below 25%, between 25 and 50%, between 50 and 75%, and above 75% indicated no heterogeneity, moderate, high, and extreme heterogeneity, respectively. Since there was substantial heterogeneity, the pooled SMD and corresponding 95% confidence intervals were calculated using a random-effects model.

The command "metandi" was used to calculate the diagnostic odds ratio (DOR), pooled specificity, specificity, positive likelihood ratio, and negative likelihood ratio. A summary receiver operating characteristic (SROC) curve was also developed.

To assess the existence of possible publication bias, the relationships between the size of the study and the magnitude of effect were assessed using Egger's regression asymmetry test at the *p* < 0.05 level of significance. Stata 14 was used for statistical analysis (STATA Corp., College Station, TX, USA). Last but not least, our research complied completely with the Preferred Reporting Items for Systematic Reviews and Meta-Analyses (PRISMA) guidelines.

## Results

### Search results and included studies

**Fig. 1:**
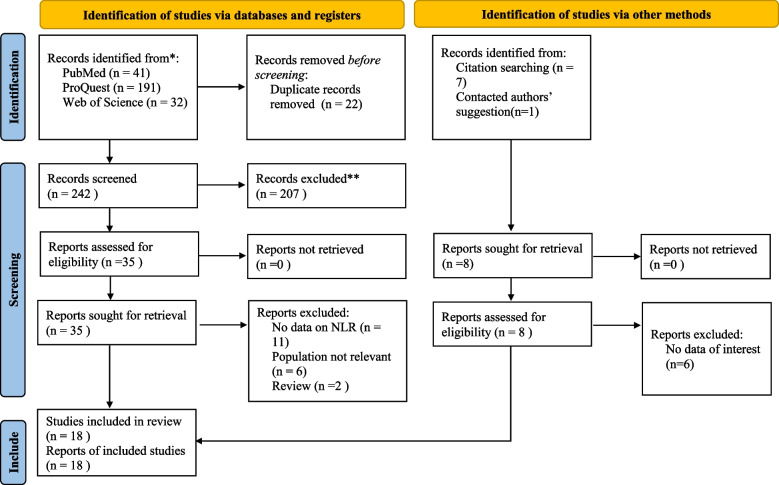
PRISMA 2020 Flow diagram for new systematic reviews which includes searches of databases, registers and other sources

In the initial search, we found a total of 272 results, and among them, 18 papers were included after multiple stags of screening [22–39], shown in Fig. 1.

### Characteristics of the population and quality assessment

In total, 18 articles were included in the analysis [22–39], including 2507 patients with endometriosis and 2179 healthy controls. Seventeen of the included studies compared patients with endometriosis with healthy controls [22–36, 38, 39], five of them compared patients with endometriosis and those with other benign tumors [25, 26, 29, 34, 38], three of them compared endometriosis patients with and without deep lesions [30, 31, 37], two of them compared endometriosis patients with and without peritoneal lesions [31, 37], four study compared endometriosis patients with and without endometrioma [30, 31, 37, 39], and four studies compared patients with stage III and IV endometriosis with those with stage I and II endometrioma [31, 32, 36, 37]. Six studies conducted receiver operating characteristic (ROC) curve analysis [25, 26, 32, 35, 36, 38]. Four of them were prospective [28, 29, 31, 32] and others were retrospective. Five of them were on East Asian patients [23–25, 29, 38] and others were on Caucasian patients. Check out Table 1 for overall characteristics and quality scores of the included articles. Table 2 shows the exclusion criteria of included studies.

**Table 1:** General characteristics of included studies

Author	Year	Country	Design	Ethnicity	Endometriosis		Healthy control		Other benign tumors		Type				Stage				Cut off point	SN	SP	NOS score
															Stage I/II		Stage III/ IV					
					N	NLR	N	NLR	N	NLR	Type of endometrioma		N	NLR	N	NLR	N	NLR				
Cho	2008	South Korea	Retrospective	East Asian	231	2.66 ± 0.34	384	1.99 ± 0.14	145	2.31 ± 0.32	_	_	_	_	_	_	_	_	2.01	60	60	7
Hua	2013	China	Retrospective	East Asian	197	2.30 ± 0.25	112	1.68 ± 0.14	102	1.93 ± 0.19	_	_	_	_	_	_	_	_	1.82	58	65	7
Sayan	2013	Turkey	Prospective	Caucasian	50	5.28 ± 2.28	50	5.06 ± 2.87	_	_	_	_	_	_	18	3.41 ± 1.38	32	5.60 ± 2.47	2.19	76	82	6
Yavuzcan	2013	Turkey	Retrospective	Caucasian	61	2.45 ± 1.73	33	2.11 ± 0.86	_	_	Endometrioma	Yes	33	2.40 ± 2.04	_	_	_	_	_	_	_	6
												No	28	2.51 ± 1.37								
Tokmak	2015	Turkey	Retrospective	Caucasian	467	2.80 ± 2.00	340	1.70 ± 0.50	_	_	_	_	_	_	_	_	_	_	1.9	70	74	8
Li	2016	China	Prospective	East Asian	100	2.43 ± 0.29	100	1.48 ± 0.52	60	1.64 ± 0.65	_	_	_	_	_	_	_	_	_	_	_	8
Vigano	2017	Italy	Retrospective	Caucasian	_	_	_	_	_	_	Deep lesions	Yes	69	2.17 ± 1.68	45	2.43 ± 2.19	124	2.08 ± 0.95	_	_	_	6
												No	145	2.16 ± 1.25								
											Peritoneal lesions	Yes	58	2.10 ± 0.98								
												No	145	2.2 ± 1.25								
											Endometrioma	Yes	98	2.08 ± 1.01								
												No	145	2.16 ± 1.25								
Seckin	2018	Turkey	Retrospective	Caucasian	267	2.30 ± 1.30	235	2.10 ± 1.20	_	_	_	_	_	_	_	_	_	_	_	_	_	8
Kalem	2019	Turkey	Retrospective	Caucasian	128	2.21 ± 0.80	_	_	85	1.66 ± 0.47	_	_	_	_	_	_	_	_	1.73	67	66	8
Targut	2019	Turkey	Retrospective	Caucasian	121	2.18 ± 0.63	136	1.70 ± 0.59	_	_	_	_	_	_	17	1.90 ± 0.81	104	2.20 ± 0.62	1.60	88	45	7
Tas	2019	Turkey	Retrospective	Caucasian	37	2.47 ± 1.01	40	2.05 ± 0.64	36	5.20 ± 3.68	_	_	_	_	_	_	_	_	_	_	_	6
Biyik	2020	Turkey	Retrospective	Caucasian	45	1.93 ± 0.78	42	1.84 ± 0.65	_	_	_	_	_	_	_	_	_	_	_	_	_	6
Karadadas	2020	Turkey	Retrospective	Caucasian	71	2.38 ± 0.14	77	2.37 ± 0.14	_	_	_	_	_	_	_	_	_	_	_	_	_	7
Moini	2020	Iran	Retrospective	Caucasian	217	5.50 ± 3.70	104	3.30 ± 2.90	_	_	Deep lesions	Yes	33	4.30 ± 2.90	_	_	_	_	_	_	_	7
												No	104	3.30 ± 2.90								
											Endometrioma	Yes	184	5.70 ± 4.00								
												No	104	3.30 ± 2.90								
Ottolani	2020	Italy	Prospective	Caucasian	324	2.21 ± 0.95	284	2.05 ± 1.50	_	_	Deep lesions	Yes	69	2.12 ± 0.95	85	2.06 ± 0.81	239	2.25 ± 0.99	_	_	_	7
												No	255	2.23 ± 0.93								
											Peritoneal lesions	Yes	41	2.04 ± 0.78								
												No	283	2.2 ± 0.96								
											Endometrioma	Yes	41	2.04 ± 0.78								
												No	110	2.090.88								
Chen,T	2021	China	Retrospective	East Asian	137	1.69 ± 0.69	137	1.59 ± 0.47	_	_	_	_	_	_	_	_	_	_	_	_	_	7
Chen,Z	2021	China	Retrospective	East Asian	31	2.22 ± 0.93	95	2.31 ± 1.21	_	_	_	_	_	_	_	_	_	_	_	_	_	6
Kedzia	2021	Poland	Prospective	Caucasian	23	4.79 ± 3.11	10	1.66 ± 1.41	_	_	_	_	_	_	_	_	_	_	_	_	_	6

*N* Number, *NLR* Neutrophil to lymphocyte ratio, *NOS* Newcastle‐Ottawa Scale

**Table 2:** Exclusion criteria of included studies

Author	Year	Exclusion Criteria	
		Factors affecting inflammatory status	Coexisting lesions (pelvic pathological conditions)
Cho	2008	_	Adenomyosis or clinical suspicion of leiomyoma
Hua	2013	Hematemesis, immune system diseases, pregnancy, abnormal liver function, tuberculosis, endocrine disease, pelvic inflammatory disease	Adenomyosis, leiomyoma
Sayan	2013	Use of hormonal medications during the 6 months prior to the laparoscopy, pregnancy, presence of chronic or acute inflammation and autoimmune disease, a presentation of pelvic inflammatory disease (PID)	Adenomyosis, leiomyoma, ovarian neoplasia
Yavuzcan	2013	Pelvic inflammatory disease, borderline ovarian tumor, patients with infectious disease, smokers, chronic or acute inflammatory disease, systemic disorder or autoimmune, malignancy	Endometrial hyperplasia, endometrial polyp, adenomyosis, myoma uteri, gynecological malignancy, benign adnexal mass
Tokmak	2015	Metabolic and autoimmune disorders, chronic inflammatory disease, or malignancy, current acute infection, inflammatory disease	Myoma uteri, adenomyosis, endometrial pathology
Li	2016	Malignant tumor, acute inflammation, and patients with severe endocrine disorder, patients received hormone or other endocrine therapy in three months, patients with severe underlying disease (severe kidney, liver, heart dysfunction)	Uterine glandular myopathy or uterine leiomyoma
Vigano	2017	Malignancy, taken antiplatelet drugs, steroid hormones, oral contraceptives, or other medications in the last 3 months before surgery	_
Seckin	2018	History of hormonal therapy, pregnancy, malignancy, acute infection, immune system and hematologic disorders, abnormal liver function, chronic inflammatory disease, current pelvic inflammatory disease	Gynecological malignancy, myoma uteri and adenomyosis, adnexal torsion or ruptured ovarian cysts
Kalem	2020	Receiving steroid or estrogen and/or progesterone, pregnancy, pelvic-systemic infection	Preoperative cyst rupture
Turgut	2019	Smoking, pregnancy, hormonal treatment, menopause, chronic diseases (connective tissue disorders, hematological, hypertension, cardiac, kidney or liver diseases, diabetes mellitus or prediabetes, asthma, hyperlipidemia), neoplastic disease, previous thrombosis, use of glucocorticoids, acute chronic inflammatory disorders, anticoagulants, antineoplastic agents, oral contraceptives, non-steroidal anti-inflammatory drugs	Endometrial pathology, adenomyosis, leiomyoma
Tas	2019	Autoimmune disorders, smoking, malignancy, hematological disease, diabetes, cardiovascular disease, advanced liver or kidney disease, acute or chronic inflammatory diseases, acute infectious disease within the last 3 months	_
Biyik	2020	Active infections, hormone-releasing tumor, autoimmune disease, inflammatory disease, dyslipidemia, cancer, diabetes, cardiovascular disease), patients using (dyslipidemia drug oral contraceptive, gonadotropin hormone releasing hormone analogue, dienogest, antihypertensive, antiplatelet drug, antidiabetic drug, steroid, anticoagulant)	_
Karadadas	2020	malignancy, pregnancy, history of endometriosis, known chronic or acute inflammation	_
Moini	2020	moking, infectious diseases, any malignancies, acute or chronic inflammatory diseases	Polyp or malignant ovarian tumors, uterine myoma, adenomyosis, endometrial hyperplasia, pelvic inflammatory disease
Ottolina	2020	Autoimmune diseases, coagulation disorders, concomitant anticoagulant therapy or use of antiplatelet at the time of surgery	Ovarian or uterine malignancy,
Chen,T	2021	Taking any hormone drugs, complicated with other endocrine or metabolic or immune diseases, having a history of pregnancy within 6 months, malignant tumors	Uterine leiomyoma and adenomyosis
Chen,Z	2021	Acute inflammation, malignancy, metabolic diseases, suspected infectious disease, autoimmune disease, oral contraceptives, Hormonal therapy, pregnancy, gonadotropin-releasing hormone analogs, or any other hormonal treatment, hemostatic agents and herbal compounds and antithrombotic	Abnormal uterine bleeding in the previous,
Kedzia	2021	An uncertain family history of thrombosis, neoplasm, a prior episode of thrombosis diagnosed as acquired or inherited thrombophilia	_

### Differences in NLR Level between patients with endometriosis and healthy controls

**Fig. 2:**
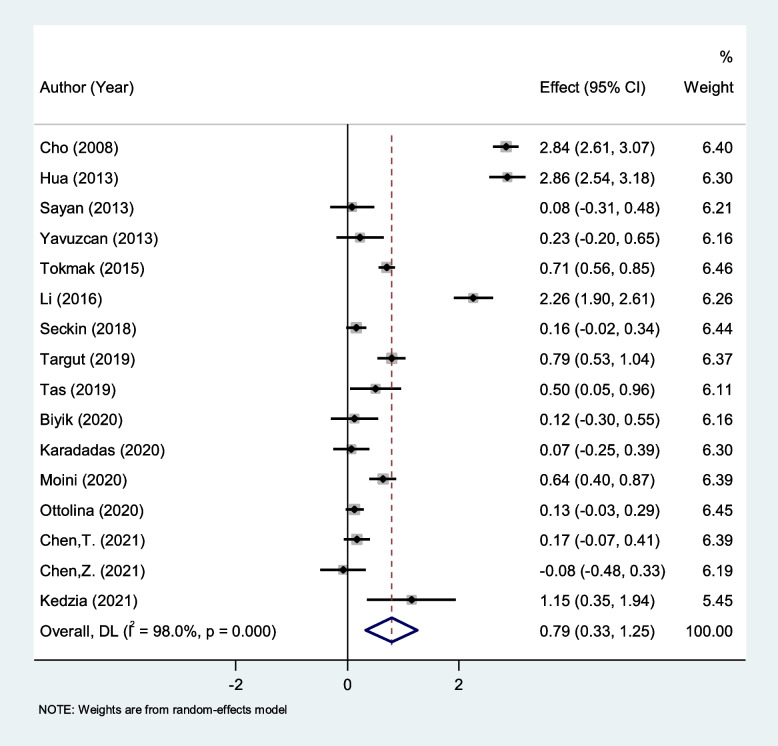
Meta-analysis of differences in NLR level between patients with endometriosis and healthy controls

Patients with endometriosis had elevated levels of NLR compared to healthy controls (SMD = 0.79, 95% CI = 0.33 to 1.25, *P* < 0.001, Fig. 2).

**Fig. 3:**
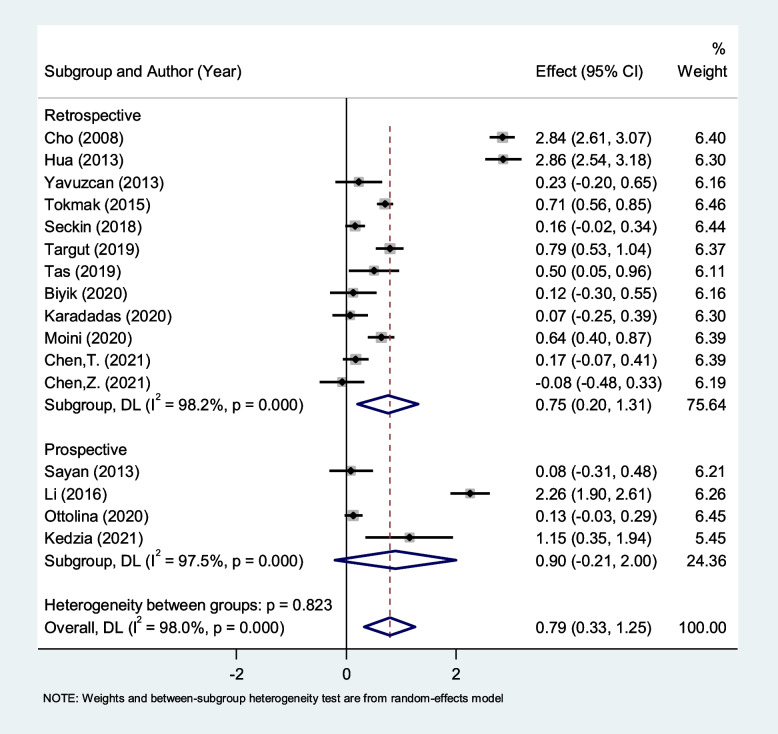
Subgroup analysis of differences in NLR level between patients with endometriosis and healthy controls, according to study design

In the subgroup analysis according to study design, we found that patients with endometriosis had elevated levels of NLR compared to healthy controls in retrospective studies (SMD = 0.75, 95% CI = 0.20 to 1.31, *P* = 0.007), but not in prospective studies (SMD = 0.90, 95% CI = -0.21 to 2.00, *P* = 0.11, Fig. 3).

**Fig. 4:**
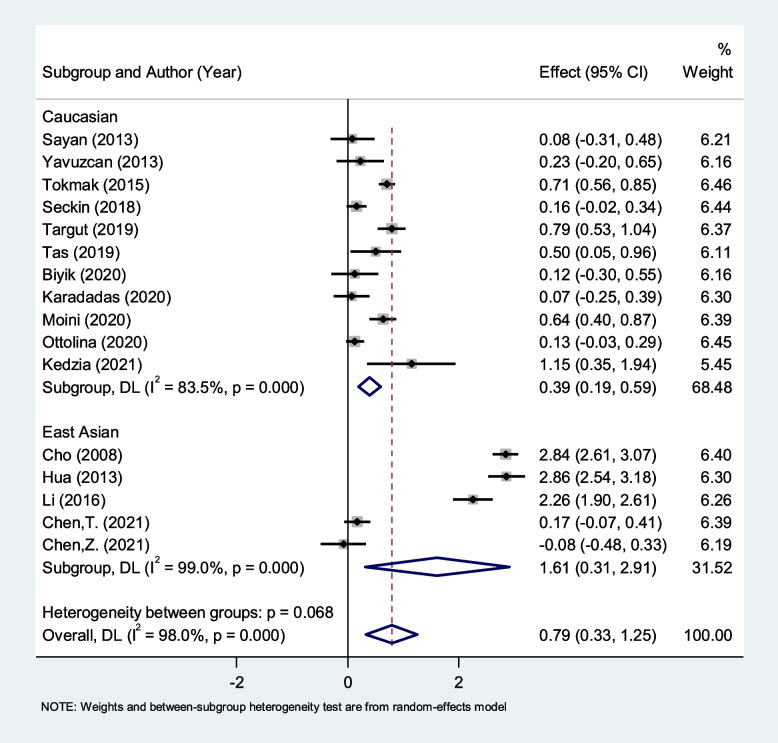
Subgroup analysis of differences in NLR level between patients with endometriosis and healthy controls, according to ethnicity

In the subgroup analysis according to ethnicity, we found that NLR is higher in patients with endometriosis compared to healthy controls among either East Asian (SMD = 1.61, 95% CI = 0.31 to 2.91, *P* = 0.015) or Caucasian (SMD = 0.39, 95% CI = 0.19 to 0.59, *P* < 0.001) ethnicity (Fig. 4).

### Differences in NLR Level between patients with endometriosis and other benign tumors

**Fig. 5:**
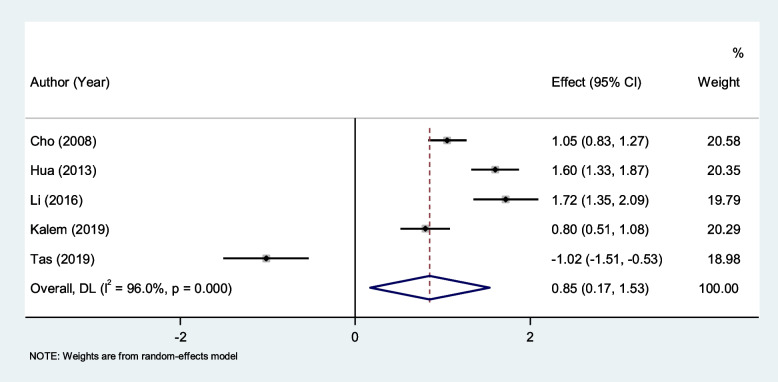
Meta-analysis of differences in NLR level between patients with endometriosis and those with other benign tumors

Patients with endometriosis had elevated levels of NLR compared to those with other benign tumors (SMD = 0.85, 95% CI = 0.17 to 1.53, *P* = 0.014, Fig. 5).

**Fig. 6:**
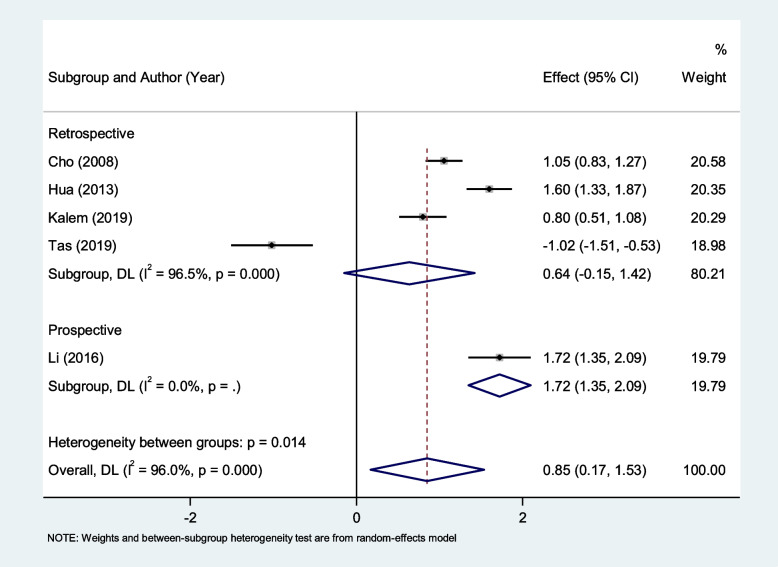
Subgroup analysis of differences in NLR level between patients with endometriosis and those with other benign tumors, according to study design

In the subgroup analysis according to study design, we found that patients with endometriosis had elevated levels of NLR compared to those with other benign tumors in prospective study (SMD = 1.72, 95% CI = 1.35 to 2.09, *P* < 0.001), but not in retrospective studies (SMD = 0.64, 95% CI = -0.15 to 1.42, *P* = 0.11, Fig. 6).

**Fig. 7:**
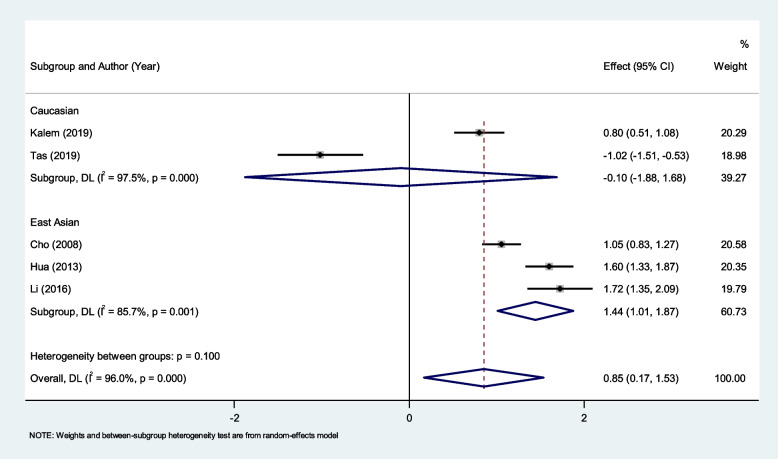
Subgroup analysis of differences in NLR level between patients with endometriosis and those with other benign tumors, according to ethnicity

In the subgroup analysis according to ethnicity, we found that NLR is higher in patients with endometriosis compared to those with other benign tumors among East Asian ethnicity (SMD = 1.44, 95% CI = 1.01 to 1.87, *P* < 0.001), but not among Caucasian (SMD = -0.10, 95% CI = -1.88 to 1.68, *P* = 0.91) ethnicity (Fig. 7).

### Differences in NLR Level between patients with different types of endometriosis

**Fig. 8:**
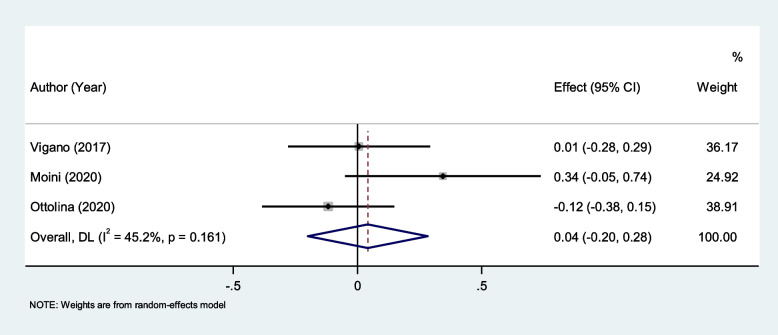
Meta-analysis of differences in NLR level between endometriosis patients with and without deep lesions

There were not any differences in NLR levels between endometriosis patients with and without deep lesions (SMD = 0.04, 95% CI = -0.20 to 0.28, *P* = 0.72, Fig. 8).

**Fig. 9:**
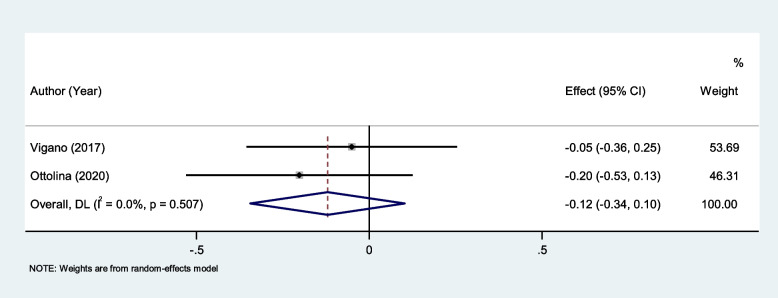
Meta-analysis of differences in NLR level between endometriosis patients with and without peritoneal lesions

NLR level was not different between endometriosis patients with and without peritoneal lesions (SMD = -0.12, 95% CI = -0.34to 0.10, *P* = 0.28, Fig. 9).

**Fig. 10:**
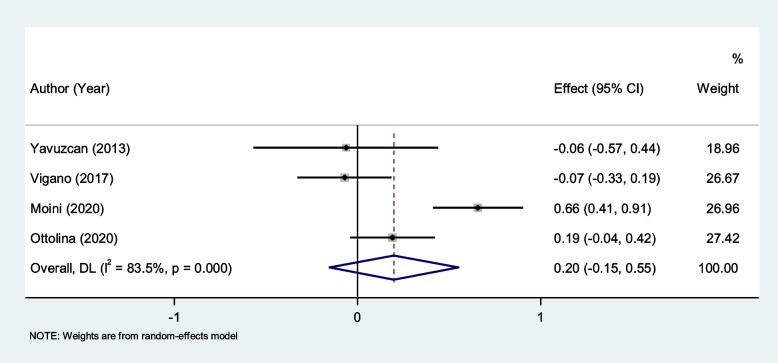
Meta-analysis of differences in NLR level between endometriosis patients with and without endometrioma

NLR level was not different between endometriosis patients with and without endometrioma (SMD = 0.20, 95% CI = -0.15 to 0.55, *P* = 0.26, Fig. 10).

### Differences in NLR Level between patients with different stages of endometriosis

**Fig. 11:**
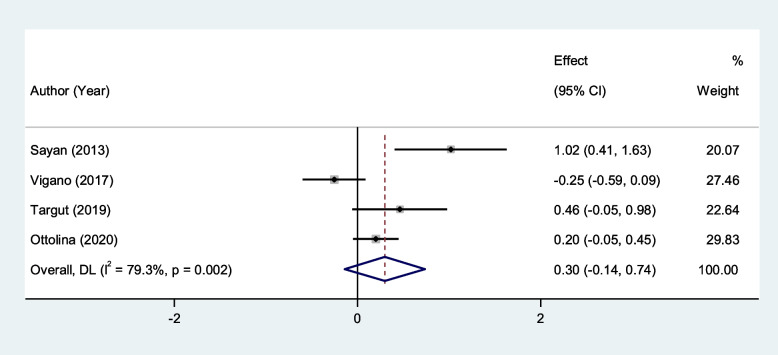
Meta-analysis of differences in NLR level between patients with stage III and IV endometriosis compared to those with stage I and II endometrioma

NLR level of patients with stage III and IV endometriosis was not different from that of patients with stage I and II endometrioma (SMD = 0.30, 95% CI = -0.14 to 0.74, *P* = 0.18, Fig. 11).

### Diagnostic value of NLR in endometriosis

**Fig. 12:**
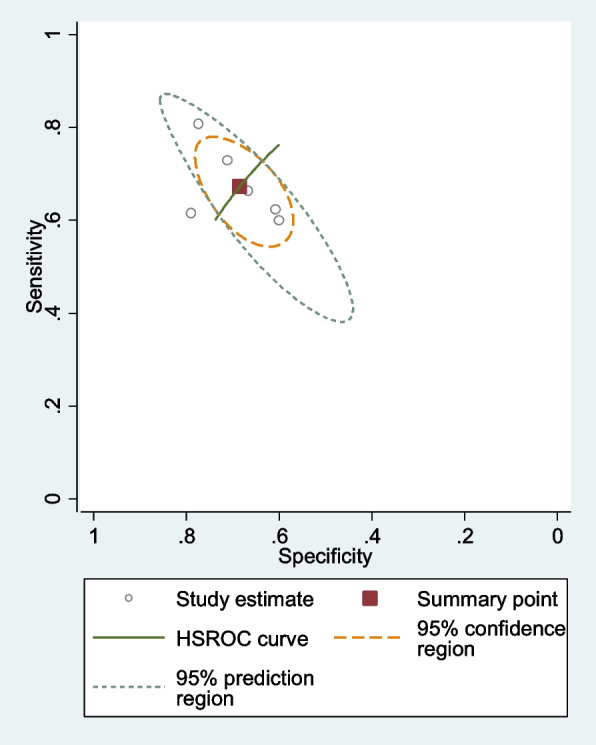
SROC curve of included studies assessing diagnostic value of NLR for endometriosis

The pooled sensitivity was 0.67 (95% CI = 0.60–0.73), and the pooled specificity was 0.68 (95% CI, 0.62–0.73). The pooled positive likelihood ratio, negative likelihood ratio, diagnostic odds ratio (DOR) of NLR were 2.13(95%CI = 1.67–2.73),0.47 (95%CI = 0.37–0.61), and 2.09 (95%CI = 1.63–2.68), respectively (Fig. 12).

### Publication bias

**Fig. 13:**
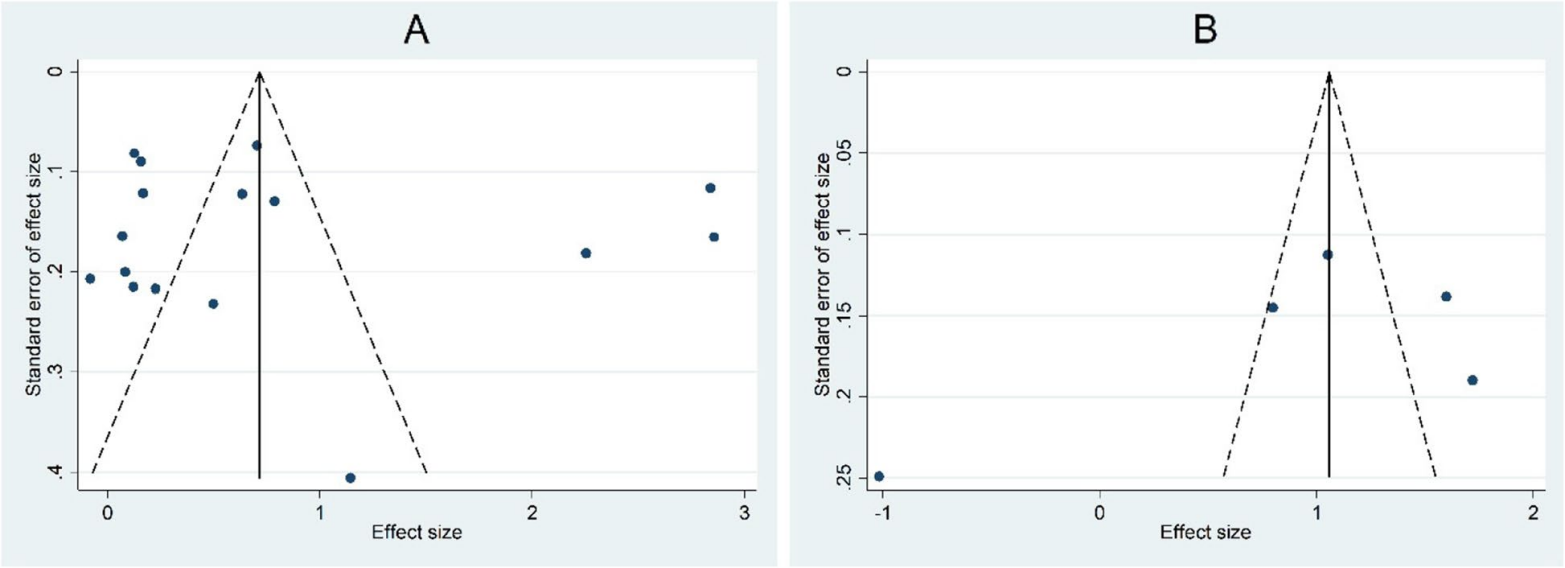
Funnel plot assessing publication bias among studies on either differences in NLR levels between patients with endometriosis and heathy controls (Figure **A**) or differences in NLR levels between patients with endometriosis and those with other benign tumors (Figure** B**)

There was no significant publication bias among studies on either differences in NLR levels between patients with endometriosis and heathy controls (Egger test *P* = 0.38, Fig. 13A) or differences in NLR levels between patients with endometriosis and those with other benign tumors (Egger test *P* = 0.42, Fig. 13B).

## Discussion

Three key conclusions emerged from our investigation. First, endometriosis patients had significantly higher NLR levels compared to healthy controls. Second, endometriosis patients of Caucasian and East Asian ethnicity exhibited higher NLR levels than healthy controls. Third, patients with endometriosis exhibited higher levels of NLR than those with other benign tumors. Appreciating the dynamic functions that neutrophils and lymphocytes play in the context of endometriosis is crucial to fully grasp the relevance of their relative proportions. A higher NLR has been reported in endometriosis patients compared to controls because of an elevation in neutrophil count and a reduction in lymphocyte count.

Infertility, pelvic discomfort, and other symptoms experienced by endometriosis patients have a negative impact on their quality of life [41, 42]. Endometriosis is a highly intricate condition that has a substantial effect on the psychological well-being, quality of life, and interpersonal connections of affected women [43, 44]. The symptoms of endometriosis gradually hinder a woman's capability to perform certain daily tasks, leading to a perception of deteriorating overall health status and well-being [45]. The repercussions of these symptoms have been incompletely explored, and additional research and clinical expertise are required to gain a comprehensive understanding of the scope of this condition. Studies have indicated that endometriosis is linked to significant direct and indirect expenses, on par with major chronic diseases like diabetes [45]. Furthermore, symptoms related to endometriosis significantly disrupt the employment of affected women, often leading to missed workdays in numerous cases [45]. New data indicates that approximately half of women who experience infertility may be affected by endometriosis [44]. The illness can impact fertility by modifying gamete transport, tuba-ovarian function, endometrial receptivity, and triggering inflammatory characteristics in peritoneal fluid, which in turn leads to changes in function and quality of sperms [46–50]. Research on oocyte donation indicates a negative impact on oocytes rather than endometrial receptivity [51]. Alongside the qualitative influence on oocytes, a quantitative decline in ovarian reserve is also a concern for women afflicted with endometriosis [52].

Significant reductions in ovarian reserve lead to unfavorable reproductive outcomes following in vitro fertilization (IVF) [53–55]. The current therapeutic approaches are far from being curative [10, 11]. Fertility preservation (FP) provides women at risk of age-related fertility decline with the opportunity of bearing children in the future using their own gametes [56]. A recent comprehensive study, the largest of its kind, presents successful outcomes achieved through oocyte vitrification for both elective FP and FP in cancer patients, while also identifying factors contributing to success [57]. Endometriosis indeed contributes to diminishing the ovarian reserve, thereby making women diagnosed with the condition increasingly eligible candidates for FP [58–60]. Currently, oocyte vitrification stands as an effective choice for elective FP in reproductive-aged women [61, 62]. In cases involving women diagnosed with endometriosis, this method additionally boasts the advantage of being relatively minimally invasive and lacking detrimental effects on ovarian reserve [59, 60].

Late diagnosis and high recurrence rates are characteristics of endometriosis [63, 64]. Although laparoscopy is the gold standard for diagnosing endometriosis, it is probably missed in cases with severe pelvic adhesions and atypical, extraperitoneal, minor lesions. Furthermore, laparoscopy has a significant risk of complications and is an expensive procedure [65]. Therefore, identifying suitable early non-invasive diagnostic markers and minimizing needless intervention methods are of significant clinical importance, and this is where NLR steps in to help.

NLR, SII, platelet-to-lymphocyte ratio (PLR), and monocyte-to-lymphocyte ratio (MLR) have been shown to correlate with endometriosis so that they might be employed as hematological markers for endometriosis diagnosis [66–68]. While using NLR for endometriosis diagnosis, the variations of this biomarker in menstrual period should be taken into account. Menstruation exhibits many features of an inflammatory process [69] and NLR as a marker of inflammation can be increased in these circumstances. In this investigation, we discovered that NLR was higher in patients with endometriosis than in healthy controls, which may properly represent the inflammatory state in endometriosis patients.

Endometriosis has been linked to a greater incidence of autoimmune inflammatory disorders (like asthma and allergy) and increased inflammatory activity [70]. This disease may develop due to high quantities of inflammatory chemokines, cytokines, and prostaglandins in the peritoneal microenvironment of endometriosis-affected women [70]. There is increasing proof that women with endometriosis have altered levels of specific subtypes of white blood cells and alternations in peritoneal fluid, endometrial and peripheral blood [71, 72]. Tokmak et al. discovered WBC alterations in endometriosis patients, including an increased neutrophil and a reduced lymphocyte count, and they validated the existence of a chronic inflammatory state in these patients [35].

These results revealed that inflammation could play a role in the development of endometriosis. TNF-alpha was one of several inflammatory cytokines that many researchers investigated. Scholl et al. [73] discovered that TNF-alpha contributes to endometriosis, and another study indicated that TNF-alpha expression was elevated in the endometriosis patients' tissues [74]. TNF-alpha may thus have a significant role in the inflammatory component of endometriosis. By starting or maintaining inflammatory reactions in the peritoneal cavity, IL-16 may also contribute to the pathogenesis of endometriosis [75]. In both luteal and proliferative biopsies, endometriosis-affected women had higher levels of chemotactic activity in macrophages, neutrophils, and inflammatory cells compared to healthy women [76]. Anti-inflammatory drugs have been evaluated as the significance of inflammation in endometriosis became clear. Mariani et al. [77] discovered that elocalcitol, a selective agonist of the vitamin D receptor, may slow the development of endometriosis in an animal model. LXA4 may inhibit the development of endometriosis in the mouse model by reducing inflammation [78].

Since inflammation is involved in endometriosis and it is acknowledged that NLR is an indicator of systemic inflammation, it may be appropriate to employ this inflammatory ratio as a diagnostic biomarker for endometriosis. However, understanding the functions of neutrophils and lymphocytes in endometriosis is necessary to offer a thorough justification for using NLR as a prognostic biomarker.

One of the important parts of innate immunity are neutrophils, which release pro-inflammatory cytokines like IL-8, vascular endothelial growth factor (VEGF), and CXCL10, which could contribute to disease progression [79–81]. Neutrophils are involved in almost all inflammatory disorders, ranging from autoimmune, acute, chronic, infectious, and non-infectious diseases [82]. Numerous investigations have revealed that neutrophils have a role in endometriosis pathogenesis [79, 83]. Regarding endometriosis, higher numbers of neutrophils have been identified in patients compared to those without the condition [84, 85]. This is most likely the consequence of elevated levels of chemotactic elements such as IL-8, human neutrophil peptide (HNP1-3), and epithelial neutrophil-activating peptide (ENA-78) [86]. It is interesting to note that estrogens have been demonstrated to affect neutrophil function [87]. It has not been confirmed yet if this characteristic plays a part in endometriosis pathogenesis. In an animal model's early phases of endometriosis, the dominancy of neutrophil infiltration into ectopic uterine tissue was observed [88, 89]. These cells' primary function is to cause inflammation by releasing IL-17A, which can promote the migration of neutrophils or other pro-inflammatory mediators such as VEGF, IL-8, and CXCL10 [83]. Recent animal research on endometriosis mice revealed that depleting neutrophils using an anti-Gr-1 antibody decreased lesion formation [90]. In this investigation, neutrophil depletion decreased the formation of the lesion in the early but not late stages of disease progression, indicating that neutrophils are required for the initial endometriosis formation [90].

T and B lymphocytes are crucial adaptative immune subsets involved in the proliferation and survival of endometrial cells. Furthermore, endometriosis is characterized by a decrease in the function of cytotoxic T lymphocytes and in the secretion of cytokines by T helper cells, and also synthesis of autoantibodies by B cells [91, 92]. Here we will discuss the role of each type of lymphocyte in endometriosis one by one.

There are several studies on the role of cellular response in endometriosis, and various features are noteworthy. Since endometriosis patients had higher quantities of cytokines indicative of Th2 lymphocytes in their peritoneal fluid and plasma, the illness was described as polarizing towards this specific cellular response [93, 94]. The normal Th2 response, which is biologically linked to healing and fibrosis, has not been thoroughly studied in the context of endometriosis [95]. The relative preponderance of Th1 cells in peripheral blood makes it difficult to interpret investigations. The CD4/CD8 ratio and the quantity of each type of cell were shown to be greater in the peritoneal fluid of affected women [96]. T-cell counts in endometrial lesions were higher than in eutopic endometrium; however, the CD4/CD8 ratio was similar.

Peripheral blood revealed no alterations [97, 98]. In contrast, Abramiuk et al.'s research showed no significant differences in the overall number of lymphocytes but a decline in the proportion of CD8 + T cells in peripheral blood [79]. Moreover, it has been discovered that the proportion of Th17 cells in a patient's peritoneal fluid increases with the illness stage [99]. It has also been shown that the endometriotic environment has higher quantities of IL-17. By promoting the generation of cytokines that cause inflammation and angiogenesis, this interleukin could accelerate the progression of endometriosis [100, 101]. Regulatory T cells, a subset of lymphocytes engaged in the cellular response, also have a crucial role in endometriosis. Although their part in the emergence of endometriosis is yet unknown, they theoretically serve a regulatory role and are implicated in the tolerance of immune cells. According to the authors of new research, there are more regulatory T cells in the patient's peritoneal fluid, which may be related to local immunosuppression that prevents the removal of ectopic endometrial cells [79]. Hanada et al. discovered a larger proportion of Treg in patients' peritoneal fluid while finding no differences in peripheral blood compared to controls [97]. Olkowska-Truchanowicz et al. provided similar findings, evaluating the percentages of CD25 + CD4 + Foxp3 + T cells in peripheral blood, which were identical to Takamura et al. [102, 103]. Another study also showed no variations in the proportion of regulatory T cells with the CD25 + CD4 + Foxp3 + phenotype in peripheral blood, indicating that their involvement is restricted to a local impact [79].

Endometriosis has long been assumed to be an autoimmune disease. Several studies demonstrated the existence of anti-endometriosis antibodies in the peritoneal fluid and serum of individuals with the condition [104, 105]. Although autoantibodies are hypothesized to promote the progression of endometriosis by sustaining inflammation and activating the immune system, there is no solid proof to back up this theory [79]. The presence of autoantibodies in patients with endometriosis is further exacerbated by comorbidities. The emergence of autoimmune and immunological disorders like psoriasis, rheumatoid arthritis, and allergies are related to endometriosis [106, 107]. The existence of autoantibodies led researchers to look into the function of B lymphocytes in the endometriosis pathophysiology [79]. Most investigations in a meta-analysis on this matter imply a connection between endometriosis and elevated percentages or stimulation of B lymphocytes. There were no variations in the numbers of B cells between patients and controls in 7 of the 22 included publications [108]. B cells also secrete cytokines like IL-6 and IL-17, which have been demonstrated to influence immunological cells like CD4 + T cells and sustain chronic inflammation [108]. Endometriosis is also related to these cytokines [86, 109, 110]. Therefore, in endometriosis, B lymphocytes may contribute to local and systemic cytokine production and inflame their microenvironment [79]. Therefore, further research is required to completely comprehend the role of B lymphocytes and their interactions with other immune cells in endometriosis disease.

The NK cell provides a connection between the adaptive and innate immune responses. Oosterlynck et al. were the first to define the role of natural killer (NK) cells in the pathophysiology of endometriosis by demonstrating that NK cell cytotoxic effect was decreased when faced with ectopic endometrial cells. This association correlated with advanced phases of the illness [89, 109, 111]. The reason behind the decreased function of NK cells in endometriosis is not fully understood. Numerous inhibitory and activating receptors were found to be abnormally expressed on their surface when compared to these cells in normal women [112–115]. This may be explained by the enhanced expression of KIR (killer cell immunoglobulin-like receptors) that has been detected in the peritumoral NK cells [116]. Chronic inflammation caused by the disease may lead to the aberrant function of NK cells [117, 118]. By removing cells that exhibit autoantigens, NK cells help maintain the balance of immunological tolerance. As a result, their decreased activity in endometriosis may account for the higher frequency of autoimmunity witnessed in the condition [119]. It has been shown that the presence of IL-6 and TGF-β in the peritoneal fluid of endometriosis-affected women decreases the cytolytic ability of NK cells [120, 121]. Furthermore, it has been shown that IL-15, which is significantly produced on endometrial stromal cells at the ectopic site, inhibits NK cell activity in vitro [122]. It was discovered that macrophages and endometrial cells interacted to lessen NK cell cytotoxicity, possibly by releasing more IL-10 and TGF-β [123].

### Strength and limitations

There are multiple features to the current meta-analysis. First, this is the first meta-analysis of the association between NLR levels and endometriosis. We discovered that patients with endometriosis had significantly higher NLR levels than healthy controls according to the pooled analysis. Furthermore, the results of subgroup analyses were mainly consistent with the main results of the study, indicating that our findings were plausible. Second, when pooling effect estimates, we employed the random-effects model, a more conservative strategy that improved the accuracy of our meta-analysis findings. Third, the publication date and language constraints were not limited for the literature search, reducing the possibility of publication bias in the findings of our study. Fourth, the majority of the included studies excluded patients with coexisting lesions, such as endometrial hyperplasia, endometrial polyp, adenomyosis, myoma uteri, gynecological, and it could strength our results. In addition, it has been reported that the level of inflammatory markers may rise in some systemic disorders and it could affect the findings. However, the majority of the included articles excluded the patients with diseases affecting inflammatory markers, including hematological disorders, acute or chronic infectious, hepatic insufficiency, renal dysfunction, steroid therapy, inflammatory diseases, or malignancies. Obviously, this exclusion criterion among included studies could increase the validity our results substantially.

There are some limitations that should be addressed. The majority of the patients are from Asia, and this could be a bias. In other words, the sample was almost representative of the Asian population but would tend to miss people from other continents. Further studies are needed to determine the association between NLR and endometriosis in other continents. Although we found that there was no association between NLR and grade and degree of migration of endometriosis, these results need to interpreted with caution because of limited number of included studies. More research on this topic needs to be undertaken before the relationship between NLR and grade and degree of migration of endometriosis is more clearly understood. In addition, we could not compare the NLR level before and after treatment of endometriosis, due to the lack of original studies on this context. further studies, which take these variables into account, will need to be undertaken.

## Conclusion

The results of this systematic review and meta-analysis demonstrate the significantly higher levels of NLR in patients with endometriosis compared to healthy controls. As a result, NLR might be utilized in clinics as a possible predictor to help clinicians diagnose endometriosis in women. Additional research is required to carry out a meta-analysis with a greater number of included publications in order to obtain more precise findings.

## Data Availability

All data generated or analyzed during this study are included in this published article.

## Abbreviations

SMD: Standardized mean difference
CI: Confidence interval
NLR: Neutrophil to lymphocyte ratio
NOS: Newcastle-Ottawa scale
DOR: Diagnostic odds ratio
SROC: Summary receiver operating characteristic
ROC: Receiver operating characteristic
PLR: Platelet-to-lymphocyte ratio
MLR: Monocyte-to-lymphocyte ratio
VEGF: Vascular endothelial growth factor
HNP1-3: Human neutrophil peptide
ENA-78: Epithelial neutrophil-activating peptide
NK: Natural killer
KIR: Killer cell immunoglobulin-like receptors
